# Exploring Novel Frontiers in Cancer Therapy

**DOI:** 10.3390/biomedicines12061345

**Published:** 2024-06-18

**Authors:** Adrian Bogdan Tigu, Ciprian Tomuleasa

**Affiliations:** 1 Medfuture Research Center for Advanced Medicine, Iuliu Hatieganu University of Medicine and Pharmacy, 400337 Cluj-Napoca, Romania; 2 Department of Hematology, Iuliu Hatieganu University of Medicine and Pharmacy, 400349 Cluj-Napoca, Romania; 3 Department of Hematology, Ion Chiricuta Clinical Cancer Center, 400015 Cluj-Napoca, Romania

Cancer progression and initiation are sustained by a series of alterations in molecular pathways because of genetic errors, external stimuli and other factors, which lead to an abnormal cellular function that can be translated into uncontrolled cell growth and metastasis. Cancer is characterized by several key hallmarks that define the progression of tumor cells and their behavior. These hallmarks include the following: induced angiogenesis, activated invasion and metastasis, sustained proliferation signaling, enabled replicative immortality, resistance to cell death and a pronounced ability to evade growth suppressors [1].

Different therapies can block growth factor receptors and reduce proliferative signaling which leads to apoptosis, necrosis or other types of cell death [2,3,4]. On the other hand, other therapies may restore the function of suppressor genes like p53 or can mimic their action and trigger cell death or can act as inhibitors for anti-apoptotic molecules, tipping the balance towards pro-apoptotic signals [5,6,7]. Furthermore, in some cases, the telomerase inhibitors can act as senescence and apoptosis promoters in telomerase dependent tumors [8,9]. Cell death can be triggered by starvation, thus anti-angiogenic therapies like VEGF inhibitors can disrupt nutrient apport to cancer cells and trigger cell death mechanisms [10,11,12]. Tumor cell vulnerabilities can also be triggered by disrupting their metabolic pathways, by inhibiting specific energetic pathways such as glycolysis or the tricarboxylic acid cycle [13,14,15].

By triggering specific hallmarks, targeted therapies can disrupt the survival mechanisms in tumor cells and trigger cell death. This Special Issue includes ten research papers and reviews that explore these approaches, showcasing innovative research on novel therapeutic compounds that exploit tumor cell vulnerabilities.

Pralea et al. delve into the metabolic reprogramming inherent in cancer cells, targeting the de novo serine biosynthetic pathway and proposing it as a potential therapeutic vulnerability on triple negative breast cancer cells. Through meticulous analysis, the research paper presents the cytotoxic potential of phosphoglycerate dehydrogenase inhibition across triple negative breast cancer cells, offering insights into potential novel therapies [16].

Genetic landscapes play a crucial role in tumor development. A comprehensive analysis uncovers the multifaced involvement of solute carrier family 31 member 1 (SLC31A1) in different tumor types. The research group meticulously explored its role as a potential therapeutic target and biomarker, evaluating its implications for cancer prognosis and treatment [17].

The intracellular signaling pathways are critical for tumor invasion and proliferation. Neural calcium sensor 1 (SCS1) was evaluated for its involvement in intracellular signaling, and as the research group highlighted, NCS1 plays a significant role in tumor progression and metastasis, offering potential avenues for novel therapies [18].

Kaynak et al. evaluated a non-contact electric field (EF) stimulation system that may modulate cancer biomarkers in normal and tumor cell lines. Their groundbreaking findings unveil EF’s potential in triggering cell cycle arrest and its potential role in intracellular signaling modulation, offering a glimpse into novel therapeutic approaches [19].

Aiming to discover potential antitumor compounds, Belosludtsev et al. evaluated Betulinic acid (BA) conjugates for their cytotoxic effect on breast adenocarcinoma and fibroblast cell lines. The research group highlighted the BA potential mechanism of action, offering insights for a novel therapeutical approach using natural compounds as antitumor agents [20]. By following the same approach, and investigating different flavones from *Citrus* spp., their anti-leukemic properties were investigated, evaluating their potential interaction with SIRT2. The findings offer a valuable insight into flavones interaction with SIRT2 and may lead to the development of potential anti-leukemic therapies [21].

Plant-derived compounds offer candidates for antitumor therapies. Inula viscosa and its sesquiterpene lactones (SLs) are intriguing candidates, with cytotoxic effects in neoplastic diseases. In the published review, the authors offer insights into SLs potential therapeutical activity, and highlight their potential mechanism of action [22].

The pancreatic ductal adenocarcinoma (PDAC) is very challenging for clinicians and researchers, thus alternative cell death mechanisms need to be explored in this context. A comprehensive review delves into non-apoptotic cell death mechanisms, such as ferroptosis, pyroptosis and necroptosis, in PDAC, and offers valuable insights into potential novel therapeutic strategies in PDAC which may improve patient management [23].

The intricate relationship between inflammation, obesity and the gastroenteropancreatic neuroendocrine system (GEN-NENs) is a field of interest for many research groups. Through an exhaustive review, Budek et al. highlight the complex interplay between inflammation, immune dysfunction, and adipose tissue in GEP-NEN development, paving the way for translational research and potential targets for novel therapies [24].

As myeloproliferative neoplasms (MPNs) display challenges in patient management, a comprehensive understanding of the associated comorbidities is needed. The published systematic review explores hypertension’s association with MPNs, offering valuable insights on its prevalence and impact on patient management [25].

These studies underscore the diverse avenues of cancer research, from the molecular mechanisms to therapeutical approaches, driving progress towards improved outcomes for the benefit of patients.
